# Ultra Uniform Pb_0.865_La_0.09_(Zr_0.65_Ti_0.35_)O_3_ Thin Films with Tunable Optical Properties Fabricated via Pulsed Laser Deposition

**DOI:** 10.3390/ma11040525

**Published:** 2018-03-29

**Authors:** Shenglin Jiang, Chi Huang, Honggang Gu, Shiyuan Liu, Shuai Zhu, Ming-Yu Li, Lingmin Yao, Yunyi Wu, Guangzu Zhang

**Affiliations:** 1 School of Optical and Electronic Information, Engineering Research Center for Functional Ceramics, Ministry of Education, Huazhong University of Science and Technology, Wuhan 430074, China; jslhust@gmail.com (S.J.); huangchix@gmail.com (C.H.); zhushuaihust@163.com (S.Z.); zhanggz@hust.edu.cn (G.Z.); 2 State Key Laboratory of Digital Manufacturing Equipment and Technology, Huazhong University of Science and Technology, Wuhan 430074, China; hongganggu@hust.edu.cn (H.G.); shyliu@hust.edu.cn (S.L.); 3 School of Physics and Electronic Engineering, Guangzhou University, Guangzhou 510006, China; emmiyao@163.com; 4 Department of Energy Materials and Technology, General Research Institute for Nonferrous Metals, Beijing 100088, China; wuyunyi_80@163.com

**Keywords:** thin films, pulsed laser deposition, deposition temperatures, annealing oxygen pressures, reflectance

## Abstract

Ferroelectric thin films have been utilized in a wide range of electronic and optical applications, in which their morphologies and properties can be inherently tuned by a qualitative control during growth. In this work, we demonstrate the evolution of the Pb_0.865_La_0.09_(Zr_0.65_Ti_0.35_)O_3_ (PLZT) thin films on MgO (200) with high uniformity and optimized optical property via the controls of the deposition temperatures and oxygen pressures. The perovskite phase can only be obtained at the deposition temperature above 700 °C and oxygen pressure over 50 Pa due to the improved crystallinity. Meanwhile, the surface morphologies gradually become smooth and compact owing to spontaneously increased nucleation sites with the elevated temperatures, and the crystallization of PLZT thin films also sensitively respond to the oxygen vacancies with the variation of oxygen pressures. Correspondingly, the refractive indices gradually develop with variations of the deposition temperatures and oxygen pressures resulted from the various slight loss, and the extinction coefficient for each sample is similarly near to zero due to the relatively smooth morphology. The resulting PLZT thin films exhibit the ferroelectricity, and the dielectric constant sensitively varies as a function of electric filed, which can be potentially applied in the electronic and optical applications.

## 1. Introduction

Recently, the ferroelectric materials have been investigated for optical applications to improve response time [1] or expand the work range to broadband [2], since the electric field-induced alteration of ferroelectric polarization can interact to polarized light [3,4]. Among the ferroelectric materials, the lanthanum-modified lead zirconate titanate with the high transparency and excellent electro-optic properties [5] has been applied in various devices, such as optical switches [6], optical shutters [7] and electro-optic modulators [8]. In the past years, the study interest was mainly on two-dimensional (2-D) PLZT thin films as a result of their potential applications for integrated optical devices, which could be because of the relatively higher electro-optic effect [9], smaller applied voltage [10] and higher speed [11] of the ferroelectric thin films than those of the ferroelectric bulk (3-D) materials. To minimize the light loss and maintain the electric properties for integrated optical devices, it is crucial to achieve high uniformity, crystalline quality and ferroelectricity of PLZT thin films even in nano-scale. Therefore, how to fabricate the high quality PLZT thin films with a low light consumption and stable ferroelectricity has become a key issue of the performance optimization for the related devices, which has been rarely reported up to date. PLZT thin films can be commonly obtained via various approaches, including chemical solution deposition [12], sol-gel processes [13] and RF magnetron sputtering [14]. Among those methods, owing to the precise stoichiometry, variable background gas, and high ablation energy, the pulsed laser deposition (PLD) can be an appropriate technique for the high quality PLZT thin films fabrication [15,16,17]. On the other hand, the severe surface morphological deterioration can gradually occur during the growth of the thin films due to the accumulated strain due to the lattice mismatch between substrate and thin films [18]. Thus, MgO with high thermal resistance, less lattice mismatch with PLZT, and high transmittance can be suitable as the substrate for PLZT thin film growth to diminish the strain between thin films and substrate, prevent the substrate diffusing, and minimize light loss.

In this work, we systematically investigate the effect of deposition temperature and annealing oxygen pressure on the phase structure, surface morphology and optical properties of PLZT thin films epitaxially grown on MgO (200) by PLD. PLZT thin films sensitively evolve as a function of the deposition temperatures owing to the enhanced nucleation with a favorable surface diffusion of adatoms. The annealing oxygen pressure also plays an essential role for the crystallization of PLZT thin films due to the correspondingly varied amounts of oxygen vacancy. Consequently, the optical properties inherently develop along with the surface morphological evolutions controlled by the variations of the deposition temperatures and annealing oxygen pressures. PLZT thin films with uniform surface and compact structure also show the ferroelectricity and electric field dependent dielectric constant at room temperature.

## 2. Methods

In this work, Pb_0.865_La_0.09_(Zr_0.65_Ti_0.35_)O_3_ (PLZT) thin films were epitaxially obtained on MgO (200) substrate with a variation of deposition temperatures and annealing oxygen pressures in the PLD system. LZT target was prepared by the hot-press sintering technique [19,20]. The target was precalcined at 900 °C for 2.5 h, and subsequently sintered at 1150 °C for 1 h for the crystallization in a sealed environment under the axial pressure of 100 MPa. Then, the target was annealed and oxidized at 850 °C for 2.5 h in a natural environment. PbO was 6 wt % excess to sustain the Pb content in the target during the sintering process.

The sample was fabricated in a PLD chamber with the certain distance between target and substrate of 55 mm. The target was excited by a KrF laser (λ_E_ = 248 nm) with a laser fluence of 1.7 J/cm^2^ at a repetition rate of 3 Hz. To systemically control the thickness, the deposition rate was previously calibrated, as shown in Figure S3 (Supplementary Materials), and each PLZT thin film was deposited for 70 min at the rate of 1.44 nm/min. Each sample was mounted on the platinum-rhodium alloy holder with the silver pastes and heated at the ramping rate of 40 °C/min under a vacuum of 6 × 10^−5^ Pa. To investigate the temperature effects, the deposition temperatures were methodically varied between 650 and 750 °C under the oxygen pressure of 30 Pa, and samples were subsequently annealed in situ for 30 min under the oxygen pressure of 50 Pa. To investigate the oxygen pressure effects, each sample was fabricated at the fixed deposition temperature of 750 °C under the oxygen pressure of 30 Pa, and then annealed with a variation of annealing oxygen pressures between 30 and 100 Pa. Finally, the samples were directly cooled down to room temperature under the oxygen environment to minimize the oxygen loss in the resulting thin films. To verify the electric properties of PLZT thin films, we chose the Nb-doped SrTiO_3_ (0.7 wt %) as the conductive substrate to fabricate PLZT thin films with the optimum process.

The phase structure of the PLZT target and thin films was characterized by X-ray diffractometer (XRD) (XRD-7000S, Kyoto, Japan) by using Cu Kа radiation in the range of 20–70°. Scanning electron microscopy (SEM) (Hitachi SU8010, Tokyo, Japan) was utilized for the morphological characterization of the target and thin films with an acceleration voltage of 15 kV. The surface roughness of each sample was obtained by atomic force microscopy (AFM) (Demension EDGE, Santa Barbara, CA, USA) under a non-contact (tapping) mode. The cation structure of the thin films was verified by X-ray photoelectron spectroscopy (XPS) (Escalab250Xi, Waltham, MA, USA). The reflectance of the thin films was carried out with a UV-VIS-NIR spectrophotometer (UV 3600 Plus, Kyoto, Japan) in the wavelength range of 300–1000 nm. The refractive index n(λ) and extinction coefficient k(λ) were measured with a dual rotating-compensator Mueller matrix ellipsometer (ME-L ellipsometer, Wuhan Eoptics Technology Co., Wuhan, China). The measurements were taken at an angle of 60–70° within the wavelength range of 300–1000 nm. The polarization–electric field (P-E) loops of the thin films were measured by a ferroelectric tester (Precision Premier II, Albuquerque, NM, USA). The dielectric measurements were performed by using the low frequency impedance analyzer (Agilent HP4294A, Santa Clara, CA, USA).

## 3. Results and Discussion

### 3.1. Influence of Temperature

**Figure 1:**
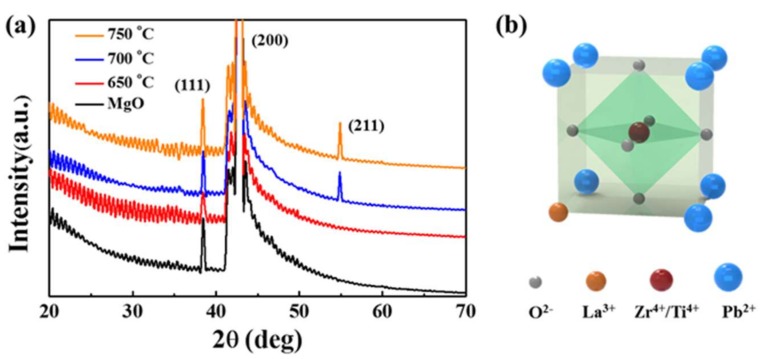
(**a**) X-ray diffraction (XRD) spectra of the pristine MgO substrate and PLZT thin films fabricated at 650, 700 and 750 °C; and (**b**) the scheme of the PLZT crystal structure.

Figure 1a shows the XRD spectra of pristine MgO substrate and PLZT thin films deposited at various temperatures between 650 and 750 °C. Figure 1b shows the schematic diagram of the PLZT crystal structure. Figure S1a shows the XRD spectra of the PLZT target in the range of 20–70°, and the surface morphology of the target are revealed in Figure S1b (Supplementary Materials). In general, the diffraction peaks at 37° and 44° were equally observed for each sample, which belong to the (111) and (200) crystal planes of MgO, respectively [21]. Meanwhile, for the thin films deposited at 650 °C, only (111) and (200) peaks of MgO can be likewise observed, as shown in Figure 1a, which could be due to the insufficient thermal energy for the adatoms to nucleate as the perovskite phase [22]. Nevertheless, as shown in Figure 1a, the (211) peak at ~55° was observed for the samples fabricated at the deposited temperatures of 700 and 750 °C, indicating the formation of the perovskite structure [23] along the preferred crystallographic orientations with low strain energy density, as similarly witnessed with the XRD spectra of the PLZT target shown in Figure S1a (Supplementary Materials). The oscillations appeared in each spectrum could derive from the avoidable instrumental noise. For the crystal structure of PLZT thin films, the La^3+^ occurred with the replacement of the Pb^2+^ sites at the corner of the perovskite structure as shown in Figure 1b. Thus, the perovskite phase can only appear at the relatively higher temperature, which was possibly because the crystallization along with the preferred orientation has been enhanced owing to the accelerated nucleation of the adatoms with more thermal energy [24]. As a result, the (211) peak was gradually increased with the elevated temperatures between 700 and 750 °C, as shown in Figure 1a. Similarly, the (110) and (200) peaks of PLZT were appeared at 32° and 44° for the thin films grown on the Si (600) at 750 °C deposited with an identical target, which could be more evidence for the formation of the perovskite phase, as shown in Figure S2 (Supplementary Materials). Regardless of the substrate, relatively weak peaks of the perovskite structure occurred, which could be induced by the much pronounced reaction between substrate and X-ray due to the much greater thickness.

**Figure 2:**
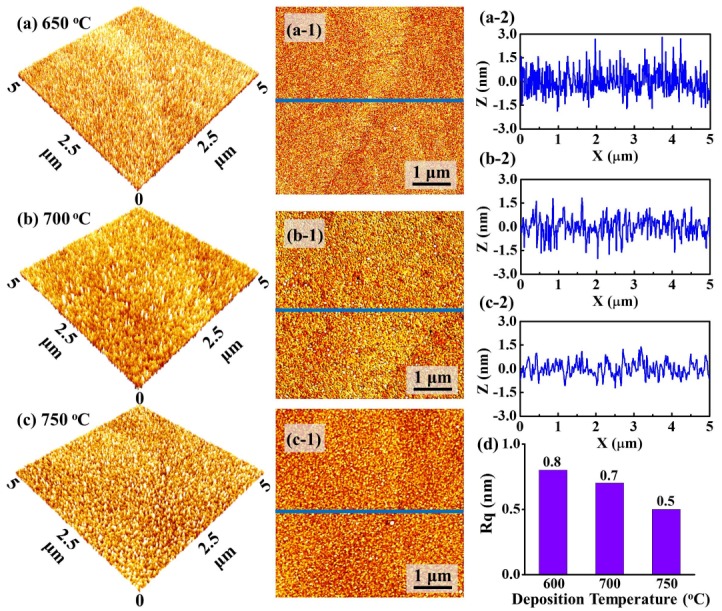
Atomic force microscopy (AFM) side-views of 5 × 5 µm^2^ for PLZT thin films on MgO (200) with various deposition temperatures: (**a**) 650; (**b**) 700; and (**c**) 750 °C; (**a-1**–**c-1**) the corresponding top-views of 5 × 5 µm^2^; (**a-2**–**c-2**) cross-sectional line-profiles acquired from the area drawn with the blue lines in (**a-1**–**c-1**); and (**d**) the root mean squared roughness (R_q_) values for PLZT thin films deposited at various deposition temperatures.

Figure 2 shows the evolution of surface morphologies of PLZT thin films deposited at various temperatures between 650 and 750 °C. Generally, PLZT thin films appeared smooth and uniform without the formation of large particles, as shown in Figure 2a–c,2a-1–c-1. Specifically, the surface morphology turned out to be relatively rough with a height modulation of ~3 nm at 650 °C, as shown in Figure 2a-2. With the increased temperature, the surface gradually became smooth, as clearly witnessed with the cross-sectional line-profiles in Figure 2b-2,c-2. The surface morphological evolution can be discussed with the thermodynamic diffusion mechanism. In a thermodynamic system, the diffusion distance of an adatom (*L_D_*) is given by $L_{D}=\;\sqrt{D_{S}t}$ [25], where *t* is the residence time of adatoms at the substrate. *D_S_* is the surface diffusion coefficient and is given by $D_{s}=D_{0}e^{\frac{-E}{kT}}$, where *D*_0_ and *E* (the diffusion barrier) are the certain values under an identical growth condition, *k* is the Boltzmann constant, and *T* is the substrate temperature (deposition temperature) [26]. Since the target was ablated by the laser with an identical power, the *t* can be comparable for each sample due to the fixed kinetic energy of the ablated atoms from PLZT. Thus, the *L_D_* can be directly determined by the deposition temperature *T*. With a wider diffusion area, the nucleation can happen uniformly at relatively high temperatures, resulting in the narrower grain boundaries and reduced surface defects [27]. As a result, the root mean squared roughness (R_q_) values of the resulting samples were correspondingly decreased from ~0.8 to ~0.5 nm as a function of the deposition temperatures, as shown in Figure 2d.

**Figure 3:**
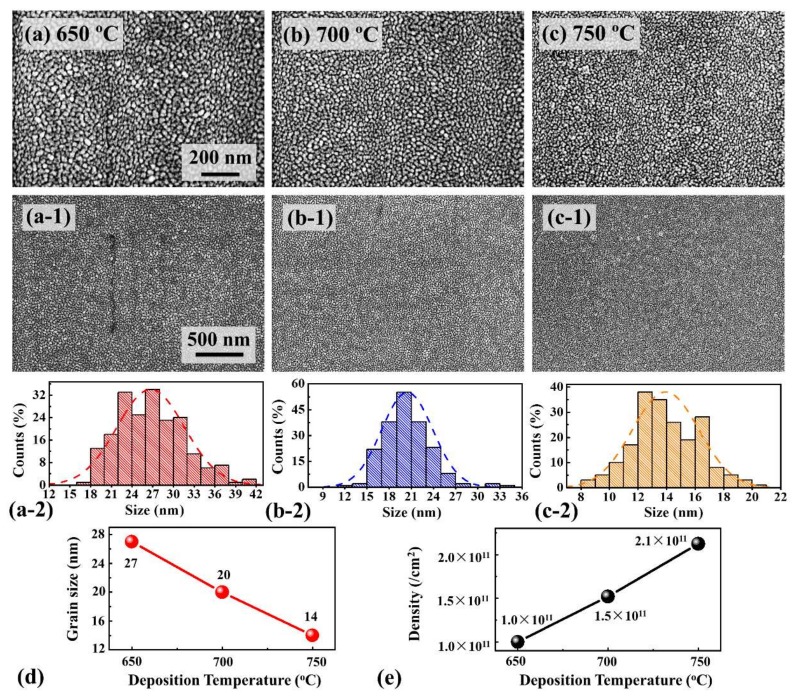
Scanning electron microscopy (SEM) images of PLZT thin films fabricated at various temperatures: (**a**) 650; (**b**) 700; and (**c**) 750 °C. SEM images are: 1.2 (*x*) × 0.8 (*y*) μm^2^ (**a**–**c**); and 2.4 (*x*) × 1.6 (*y*) μm^2^ in (**a-1**–**c-1**). (**a-2**–**c-2**) The distribution histogram and Gaussian fitting of the grain diameter of PLZT thin films; (**d**) the average grain size; and (**e**) average density of each sample.

**Figure 4:**
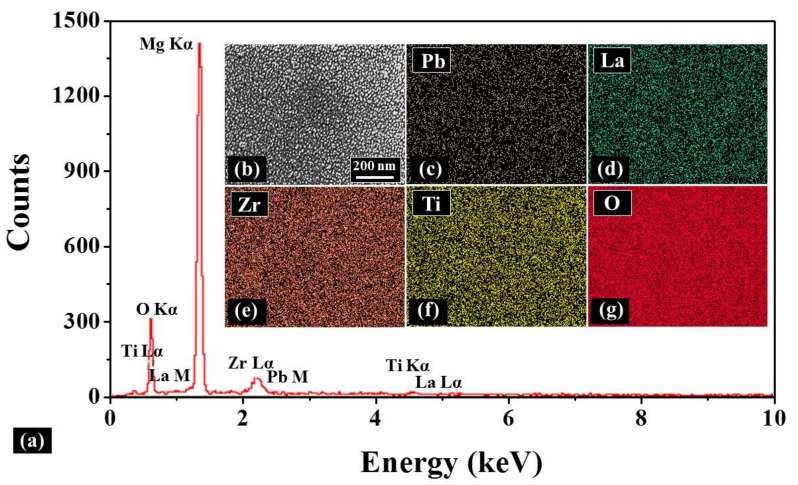
(**a**) Energy-dispersive X-ray spectroscopy (EDS) analysis of PLZT thin film deposited at 750 °C and annealed under an oxygen pressure of 50 Pa. The inset is: (**b**) SEM image of 1.04 (*x*) × 0.7 (*y*) µm^2^; and (**c**) EDS maps of: Pb; (**d**) La; (**e**) Zr; (**f**) Ti; and (**g**) O.

Figure 3 shows the effect of various deposition temperatures on the growth of the PLZT crystallite grains with an identical thin film thickness. Generally, the crystallite grains appeared compactly and uniformly with a small grain size for the samples fabricated at each deposition temperatures, as shown in Figure 3a–c,3a-1–c-1. As shown in Figure S1b (Supplementary Materials), the grains in the PLZT target synthesized via thermal press were homogeneous and compact with a grain size of several micrometers, which could potentially ensure the uniformity of the deposited thin films. For the thin films deposited at 650 °C, the grains distributed with a relatively wider size variation of between 22 and 30 nm due to the unfavorable surface diffusion of adatoms, as shown in Figure 3a,a-2. With the increased temperatures between 700 and 750 °C, the crystallite grains gradually became denser with the decreased grain size resulting from the enhanced nucleation [25,26], as shown in Figure 3b,c,b-2,c-2. Consequently, the average grain size noticeably decreased by 48% to ~14 nm with the increased deposition temperatures, and the corresponding average density increased from 1.0 × 1011 to 2.1 × 1011 cm−2, as shown in Figure 3d,e. Up to date, PLZT thin films fabricated via various methods were quite rough with the Rq over ~3 nm, and the crystallite grains were commonly over ~30 nm at least [28,29,30]. On the one hand, the PLZT adatoms obtained a comparatively high kinetic energy by the high deposition temperature and excitation laser energy, which potentially increased the nucleation sites for the crystallization [31,32]. On the other hand, the reduced deposition interval allowed a favorable surface diffusion of the adatoms, which can further improve the surface uniformity [33]. Moreover, the thickness of each sample was similar due to the fixed deposition time, which was about 100 nm, as shown in Figure S4 (Supplementary Materials). Figure 4 shows the Energy-dispersive X-ray spectroscopy (EDS) elemental analysis of the resulting PLZT thin films fabricated at 750 °C under an oxygen pressure of 50 Pa during annealing. EDS spectra are shown in Figure 4a, and the surface morphology of the corresponding sample is revealed with the SEM image in Figure 4b. Generally, the Pb, La, Zr, Ti and O were evenly observed in PLZT thin films, as shown in Figure 4c,g, indicating a uniform elements distribution throughout the whole thin films. As shown in Figure 4a, the Kα peak of O, Lα peak of Zr and M peak of Pb occurred at 0.52, 2.04, and 2.34 KeV, respectively, which can be the evidence for the formation of PLZT thin films. Similarly, the M and Lα peaks of La appeared at 0.83 and 4.65 keV, and the Lα and Kα peaks of Ti were detected at 0.45 and 4.51 keV [34].

**Figure 5:**
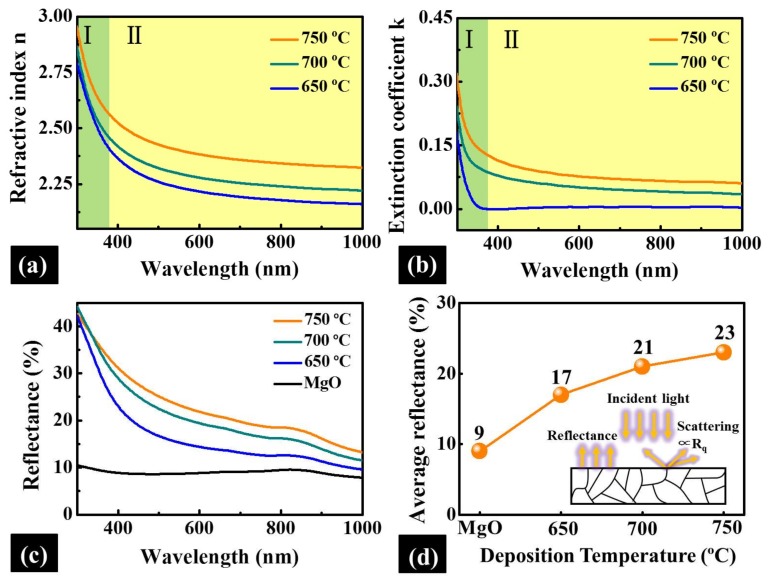
The refractive index (**a**); and extinction coefficient (**b**) of PLZT thin films deposited at various temperatures over a wavelength between 300 nm and 1000 nm. (**c**) The reflectance spectra of the pristine MgO substrate and PLZT thin films at different deposition temperatures in the visible and near-infrared regions. (**d**) Plots of the corresponding average reflectance of MgO and PLZT thin films. The inset of (**d**) is the scheme of the light attenuation of PLZT thin films.

Figure 5a,b shows the effect of different deposition temperatures on the refractive index n and extinction coefficient k of PLZT thin films over a wavelength between 300 and 1000 nm. In general, the n(λ) gradually increased along with the elevated deposition temperatures, as shown in Figure 5a. Specifically, the n(λ) changed from 2.20 to 2.37 as a function of the deposition temperatures when the wavelength of incident light was 632.8 nm, which can be ascribed to the improved surface morphology owing to the decreased grain boundaries and defects of PLZT thin films [35]. Meanwhile, for each sample, the n(λ) dispersion curves similarly decreased with the increased incident light wavelength. Noticeably, the n(λ) dispersion curves evolution can generally be divided into two phases: Phase I in the range of short wavelengths and Phase II at relatively longer wavelengths. Particularly, the n(λ) drastically decreased at the Phase I, and the decrease became slow at Phase II, which can be possibly induced by the foundational absorption in the range of band gaps [36]. Likewise, the k(λ) dispersion curves of the resulting thin films gradually increased with the increased deposition temperatures, as shown in Figure 5b. Regardless of the samples fabricated at various temperatures, the k(λ) curve radically dropped near zero at relatively low wavelengths in Phase I, as shown in Figure 5b, indicating the low absorption and scattering in the range of long wavelengths [37].

Figure 5c shows the optical reflectance spectra for MgO substrate and PLZT thin films, and the corresponding average values within a range between 300 nm and 1000 nm are summarized in Figure 5d. In general, the reflectance of each PLZT thin films increased along with the decreased wavelength between 1000 and 300 nm, as shown in Figure 5c, which was exactly in accordance with the evolution of the k [38]. As mentioned, the k was negligible at long wavelengths, and the incident light can be significantly absorbed and reflected at the relatively short wavelength due to the drastic increase in the k. Meanwhile, the reflectance of PLZT thin films gradually increased as a function of the deposition temperatures, as shown in Figure 5c, resulting in a 35% increase in the average reflectance value from 17% to 23%, as shown in Figure 5d. The inset of Figure 5d depicts the scheme of the light attenuation of PLZT thin films, and the increased reflectance can be mainly attributed to the decrease of light dissipation from surface scattering as a function of the surface roughness [39]. Commonly, the fringe can appear in the reflectance spectra for the epitaxial thin film due to the interference oscillation resulted from the phase retardation between the reflected light beams at the film-substrate interface and those at the film surface [40]. However, throughout the wavelength, the interference oscillation was not observed for any samples, which could be due to the ultra-thin thickness of the synthesized thin films in contrast with that of MgO.

### 3.2. Influence of Oxygen Pressure

**Figure 6:**
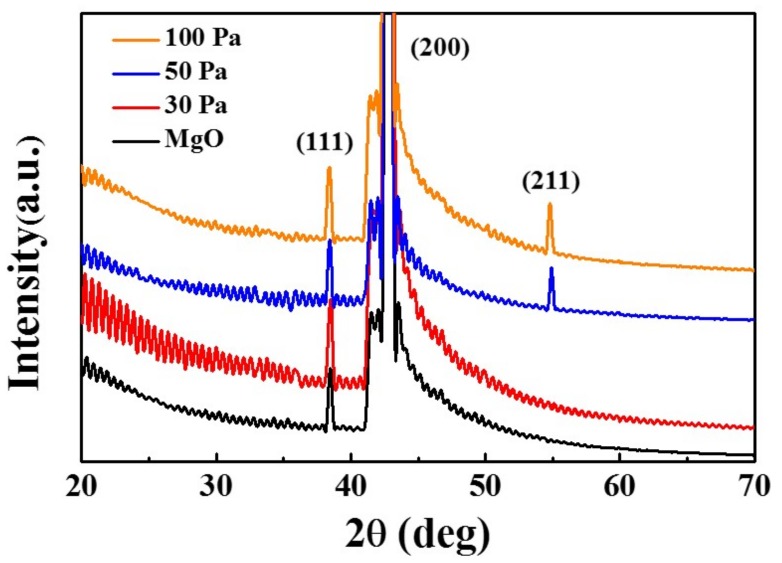
XRD spectra of MgO substrate and PLZT thin films by varying the annealing oxygen pressure at 30, 50 and 100 Pa.

Figure 6 shows XRD spectra of PLZT thin films on MgO (200) annealed at a fixed temperature 750 °C under various oxygen pressures between 30 and 100 Pa. Similar to the temperature series, the (111) and (200) of MgO were pronouncedly observed at 37° and 44°, as shown in Figure 6, and the typical perovskite structure (211) peak at ~55° gradually became noticeable with the increased oxygen pressure. The increase of the (211) peak indicated the enhancement in the preferred orientation of the thin films, which might be because the increased oxygen pressure can effectively reduce the oxygen vacancy in the thin films, and in turn improve the crystallization of the thin films even at an identical temperature [41].

**Figure 7:**
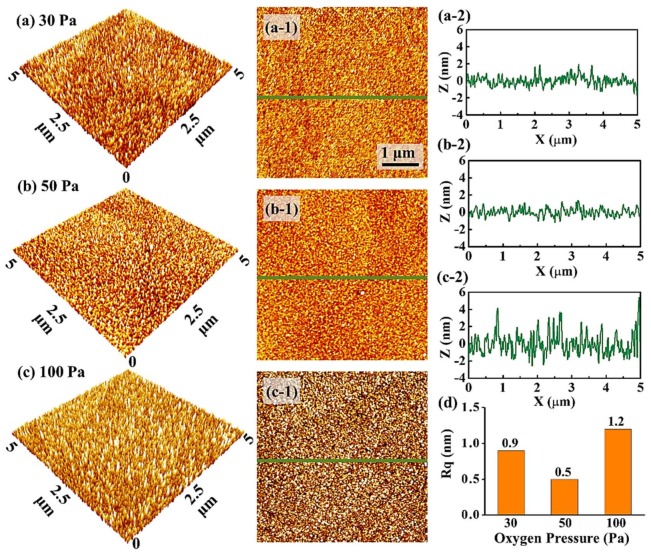
AFM side-views (5 × 5 µm^2^) of PLZT thin films fabricated on MgO (200) by varying the annealing oxygen pressure: (**a**) 30; (**b**) 50; and (**c**) 100 Pa; (**a-1**–**c-1**) the corresponding top-views of 5 × 5 µm^2^; (**a-2**–**c-2**) cross-sectional line-profiles acquired from the area drawn with the green line in (**a-1**–**c-1**); and (**d**) the R_q_ values of the samples annealed at different oxygen pressure.

Figure 7 shows the evolution on surface morphologies of PLZT thin films annealed at different oxygen pressures between 30 and 100 Pa. In general, the surface roughness decreased initially with the increased oxygen pressures from 30 to 50 Pa, and gradually increased with the further increased oxygen pressures, as shown in Figure 7a–c,7a-1–c-1. Specifically, the fluctuation of the cross-sectional line-profiles for the sample fabricated under a pressure of 30 Pa was with a modulation of ±2 nm, and modulation decreased to ±1 nm for the sample utilized under a pressure of 50 Pa, as shown in Figure 7a-2,b-2. At 100 Pa, the modulation increased to ±3 nm, and the grain size increased obviously in Figure 7c-2. Accordingly, the R_q_ of PLZT thin films was initially decreased by ~44% under annealed oxygen pressures of between 30 and 50 Pa, and increased to ~1.2 nm at 100 Pa, as shown in Figure 7d. Obviously, the oxygen pressures played a crucial role for the crystallization of PLZT thin films [42,43,44]. At relatively low pressures, many oxygen vacancies formed during the crystallization of PLZT thin films, which can consequently result in the increased surface roughness due to the formation of the defects [45]. However, at excess annealed oxygen pressure, the collision between the ablated atoms from PLZT and oxygen molecules was stronger, and, thus, a relatively weaker kinetic energy of adatoms can be expected, which eventually restrained the surface diffusion of adatoms [46]. As a result, the oversize grains were witnessed in PLZT thin films, leading to the increases in the roughness.

**Figure 8:**
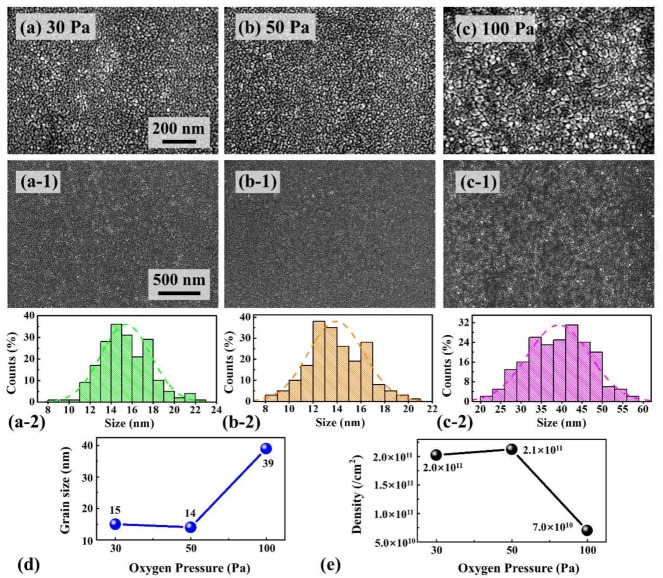
SEM images of PLZT thin films annealed at various oxygen pressure: (**a**) 30; (**b**) 50; and (**c**) 100 Pa. SEM images are: 1.2 (*x*) × 0.8 (*y*) μm^2^ (**a**–**c**); and 2.4 (*x*) × 1.6 (*y*) μm^2^ (**a-1**–**c-1**). (**a-2**–**c-2**) The distribution histogram and Gaussian fitting of the grain diameter of PLZT thin films; (**d**) the average grain size; and (**e**) the average density of each sample.

**Figure 9:**
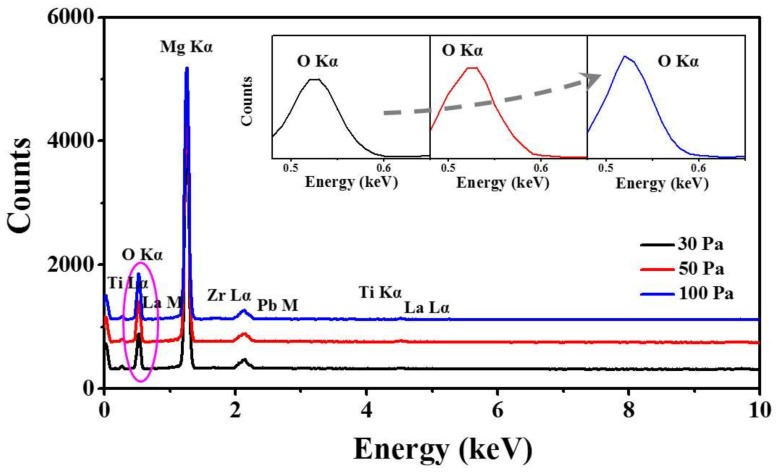
EDS analysis of PLZT thin films by varying the annealing oxygen pressure at 30, 50 and 100 Pa. The inset is the enlarged spectra for O Kα peak of each sample.

Figure 8 shows the effect of various annealing oxygen pressures on the evolution of the PLZT crystallite grains growth at a fixed annealing temperature of 750 °C. In general, PLZT thin films under each pressure formed uniformly and the surface morphology of the thin films slowly developed as a function of the oxygen pressures, as shown in Figure 8a–c,8a-1–c-1. The corresponding distribution of grain size and Gaussian fitting for each sample are shown in Figure 8a-2–c-2. When the oxygen pressure was 30 Pa, the thin films appeared crumbly with randomly distributed tiny pores around the grain boundaries, but the thin films were still smooth with the average diameter of grains 15 nm, as shown in Figure 8d. At 50 Pa, the increased oxygen pressure improves the crystallization of PLZT thin films, leading to a smaller grain size of ~14 nm and much more packed morphology with the average density of 2.1 × 10^11^ cm^−2^ as a result of reduced oxygen vacancies, as shown in Figure 8d,e. Nevertheless, at 100 Pa, the grain size visibly swelled with an average size of ~39 nm and the average density decreased to 7.0 × 10^10^ cm^−2^, which can be an obvious evidence of the overgrowth of grains in PLZT thin films. Moreover, the thickness of each sample was about 100 nm, as shown in Figure S5 (Supplementary Materials). Figure 9 shows the EDS elemental analysis of PLZT thin films annealed at the oxygen pressure between 30 and 100 Pa. Generally, all elements of PLZT thin films were detected. However, the Kα peak of O increased with the increased annealing oxygen pressure, which demonstrated the increased of the oxygen content in PLZT thin films. The XPS spectra clarified the actual chemistry changes in the thin films with varied annealed oxygen pressure, as shown in Figure S6 (Supplementary Materials). Specifically, the binding energy value of Pb 4f was decreased at relatively low oxygen pressure, and the detailed values are summarized in Table S1 (Supplementary Materials). According to the Handbook of XPS, the binding energy of 4f_7/2_ around 138.8 eV corresponds to Pb oxide (PbO) and the peak that appears near 136.7 eV corresponds to metallic Pb [47,48,49]. The shift in 4f_7/2_ peak towards lower binding energy meant the valence of Pb transformed from +2 to 0 in the thin films, with the consequent creation of many oxygen vacancies [50].

**Figure 10:**
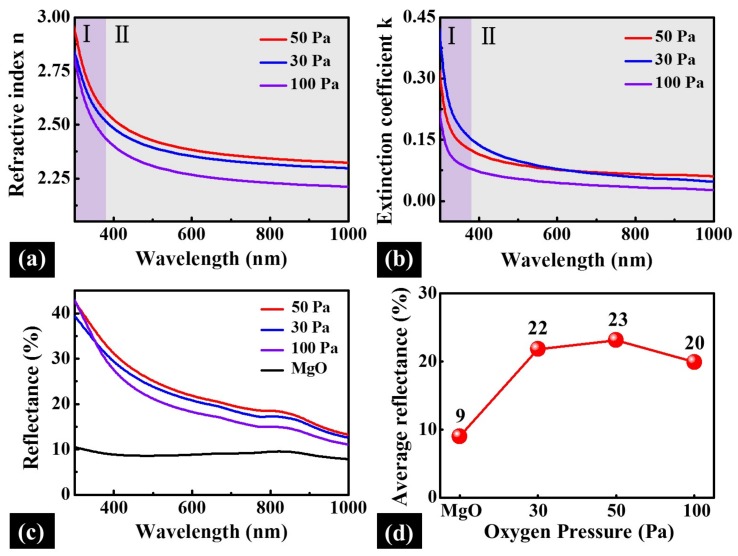
Refractive index (**a**); and extinction coefficient (**b**) of PLZT thin films annealed at various oxygen pressure as a function of wavelength in a range of 300–1000 nm. (**c**) The reflectance spectra of the MgO substrate and PLZT thin films at different annealing oxygen pressure over the wavelength range between 300 and 1000 nm. (**d**) The plot of the corresponding average reflectance of the pristine MgO substrate and PLZT thin films.

Figure 10 shows the optical properties of PLZT thin films annealed at different oxygen pressure over a wavelength range between 300 nm and 1000 nm. As shown in Figure 10a,b, all of the n(λ) and k(λ) dispersion curves decreased with the increase of incident light wavelength, and exhibited various decreasing rate at two phases due to the fundamental absorption. Specifically, with the oxygen pressure increased from 30 to 50 Pa, the refractive index relatively developed from 2.34 to 2.37 when the wavelength of incident light was 632.8 nm. Nevertheless, at 100 Pa, the refractive index sharply decreased to 2.26, possibly due to the increased roughness induced by oversize grains and defects of PLZT thin films. As shown in Figure 10b, the k(λ) radically decreased at the short wavelengths and even around zero at the long wavelengths, as observed for each sample. Consequently, PLZT thin films showed a tunable reflectance at different wavelength regions due to the varied extinction coefficient, as shown in Figure 10c. With the increased oxygen pressures, the average reflectance value slightly increased from ~22% to ~23% between 30 and 50 Pa, and dropped to 20% at 100 Pa, as shown in Figure 10d, which can be ascribed to the enhanced surface scattering resulted from the increased roughness [51].

**Figure 11:**
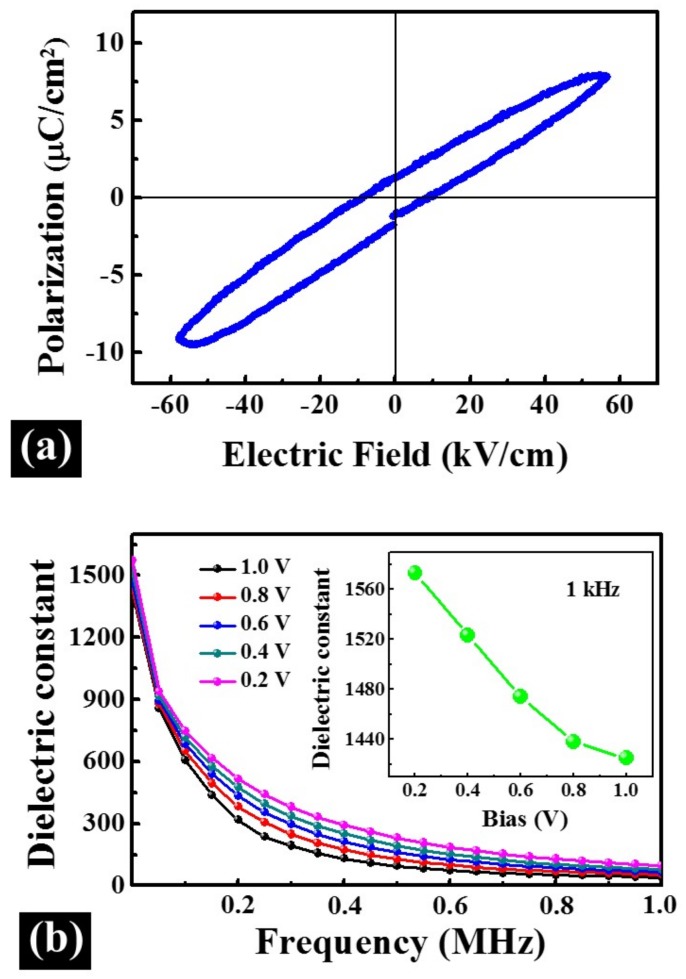
(**a**) The P-E loop; and (**b**) room-temperature frequency dependent dielectric constant of PLZT thin films fabricated at 750 °C under the oxygen pressure of 50 Pa during annealing. The inset is the dielectric constant of PLZT thin films as a function of the bias voltage with the fixed frequency of 1 kHz.

Figure 11 shows the electric properties of PLZT thin films fabricated at the 750 °C under the oxygen pressure of 50 Pa during annealing. The polarization–electric field (P-E) loop of the thin films were measured between −2 and 2 V, as shown in Figure 11a. The slim P-E loop, characteristic of relaxor-ferroelectric materials [52,53], was also observed in the PLZT target, as shown in Figure S7 (Supplementary Materials), which can be advantageous to reduce the operating voltage and energy loss [54]. The frequency dependence of dielectric constant of PLZT thin films at different bias voltages measured from 1 kHz to 1 MHz at room temperature, as shown in Figure 11b. The inset is the dielectric constant of PLZT thin films as a function of the bias voltage with the fixed frequency of 1 kHz. In general, the dielectric constant for PLZT thin films at each bias voltage decreased with the increased frequency, which can be induced by the incomplete polarization at relatively higher frequencies. Under higher frequencies, the slow polarization process, such as space charges, can limit the switch of the electric domain [55]. Specifically, the dielectric constant of PLZT thin films was more sensitively to the frequency than the bulk material, which decreased much quicker than that of the bulk material with the increased frequency, as shown in Figure S8 (Supplementary Materials). At 1 kHz, the dielectric constant of PLZT thin films clearly decreased with the increased bias voltages, as shown in inset of Figure 11b, and the detailed values are summarized in Table S2 (Supplementary Materials). The characteristic of this curve could be attributed to the constraint of the electric dipoles under an external electric field, and the contribution to the dielectric constant become weaker as the increased bias voltages [56].

## 4. Conclusions

In summary, the evolution in the surface morphologies and optical properties of PLZT thin films deposited on MgO (200) substrates via PLD is systematically investigated with various deposition temperatures and annealing oxygen pressures. The perovskite structure can only be obtained at relatively high temperatures and annealing oxygen pressures with the sufficient thermal energy supply and oxygen compensation. The elevated temperatures can effectively improve the surface uniformity and grain density owing to the increased nucleation sites with a broaden diffusion length of the adatoms. Meanwhile, the increased annealing oxygen pressure can compensate the oxygen vacancies in the films, and in turn directly determine the surface roughness and grain growth. The refractive index gradually developed from 2.20 to 3.37 (λ = 632.8 nm) as a function of the increased temperatures owing to the reduced light consumption induced by the surface scattering. Nonetheless, the refractive index initially increased with additional oxygen supply resulted from the reduced surface roughness with less oxygen vacancies formation during the crystallization of PLZT thin films, and the refractive index dropped at the higher annealing oxygen pressure due to the defects and large roughness of the film induced by the overgrowth of the crystallite grains. The best quality PLZT thin films with the lowest R_q_ can be obtained at 750 °C under 50 Pa, whose average reflectance can be radically raised by ~150% over the range of visible light to near-infrared wavelength. Meanwhile, the thin films maintained noticeable ferroelectricity and tunable dielectric constant at room temperature, which can be suitable for the optical and electrical applications with enhanced light efficiency and excellent electric properties.

## Supplementary Materials

The following are available online at http://www.mdpi.com/1996-1944/11/4/525/s1, Figure S1: (a) X-ray diffraction (XRD) spectra in a range of 20–70°; and (b) scanning electron microscopy (SEM) image of the PLZT target. SEM image is 23 (x) × 23 (y) μm2; Figure S2: XRD spectra in a range of 20–80° of PLZT thin films on Si (600) fabricated at 750 °C under an annealing oxygen pressure of 50 Pa. The inset is the spectra between 30° and 50°; Figure S3: (a) Calibration curves for the growth rate of PLZT thin films on MgO (200); and (b) atomic force microscopy (AFM) images of steps for samples deposited with different repetition times of the excitation laser: 1000, 2000, 3000 and 5000; Figure S4: SEM cross-sectional views of PLZT thin film fabricated at various temperatures: (a) 650; (b) 700; and (c) 750 °C; Figure S5: SEM cross-sectional views of PLZT thin films annealed at various oxygen pressure: (a) 30; (b) 50; and (c) 100 Pa; Figure S6: (a) X-ray photoelectron spectroscopy (XPS) spectra of PLZT thin films annealed at various oxygen pressure: 30, 50, and 100 Pa. Core level spectra of Pb 4f for each sample: (b) 30; (c) 50; and (d) 100 Pa. Figure S7: The P-E loop of the PLZT target; Figure S8: The room-temperature frequency dependent dielectric constant of the PLZT target; Table S1: Binding energy peak positions of the core levels of Pb for PLZT thin films annealed at various oxygen pressure; Table S2: The dielectric constant of PLZT thin films at each bias voltages with the fixed frequency of 1 kHz.
