# Supplementary material for: Ultra Uniform Pb0.865La0.09(Zr0.65Ti0.35)O3 Thin Films with Tunable Optical Properties Fabricated via Pulsed Laser Deposition
Source: Materials (Basel). 2018 Mar 29;11(4):525. doi: 10.3390/ma11040525 (PMC5951371; doi:10.3390/ma11040525)
Supplement: Supplementary file 1 [file materials-11-00525-s001.pdf]

# Electronic Supporting Information

# Ultra uniform $Pb_{0.865}La_{0.09}(Zr_{0.65}Ti_{0.35})O_3$ thin films with tunable optical properties fabricated via pulsed laser deposition

**Shenglin Jiang[a], Chi Huang[a], Honggang Gu[b], Shiyuan Liu[b], Shuai Zhu[a], Ming-Yu Li[a,*], Lingmin Yao [c], Yunyi Wu [d], Guangzu Zhang[a]**

**a.** School of Optical and Electronic Information, Engineering Research Center for Functional Ceramics, Ministry of Education, Huazhong University of Science and Technology, Wuhan, Hubei 430074, China.

**b.** State Key Laboratory of Digital Manufacturing Equipment and Technology, Huazhong University of Science and Technology, Wuhan, Hubei 430074, China.

**c.** School of Physics and Electronic Engineering, Guangzhou University, Guangzhou, 510006, China

**d.** Department of Energy Materials and Technology, General Research Institute for Nonferrous Metals, Beijing 100088, China .

* Corresponding author.

E-mail address: mingyuli.oliver@gmail.com

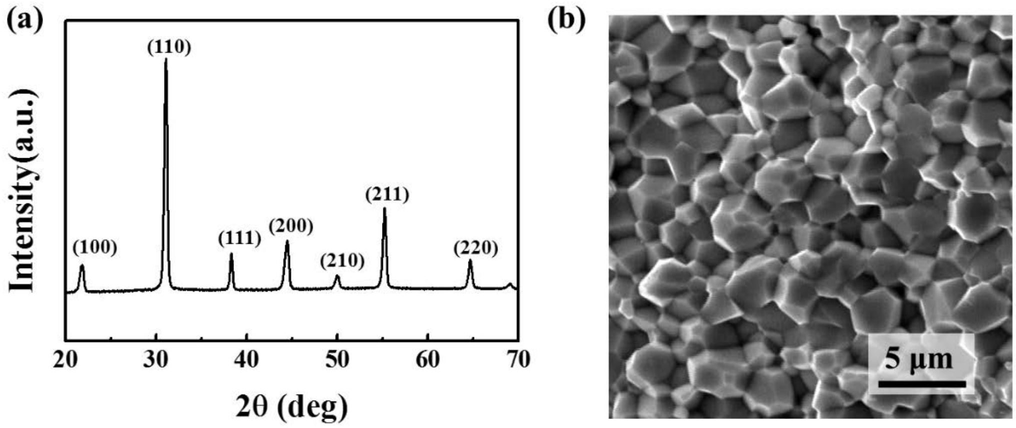


**Figure S1.** (a) X-ray diffraction (XRD) spectra in a range of 20 - 70°. (b) Scanning electron microscopy (SEM) image of the PLZT target. SEM image is 23 (x) × 23 (y) $\mu m^2$.

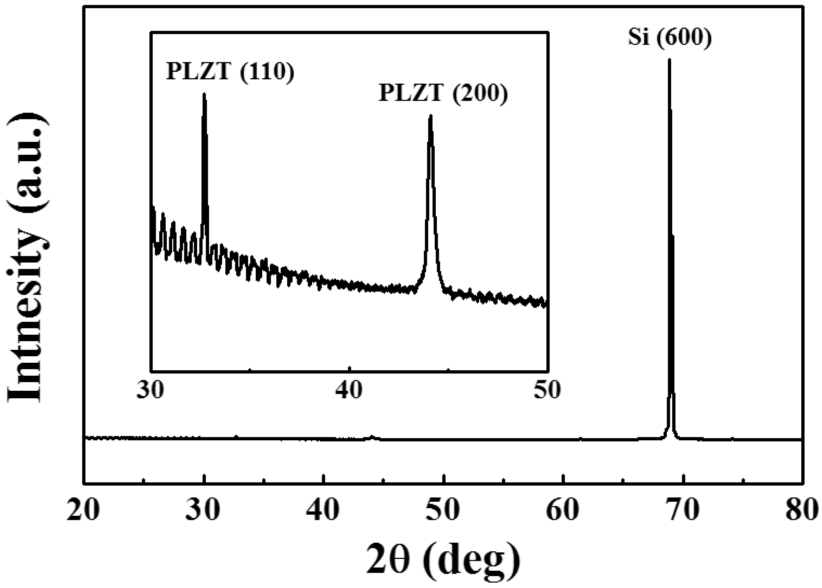


**Figure S2.** XRD spectra in a range of 20 - 80° of the PLZT thin films on Si (600) fabricated at 750 °C under an annealing oxygen pressure of 50 Pa. The inset is the spectra between 30° to 50°.

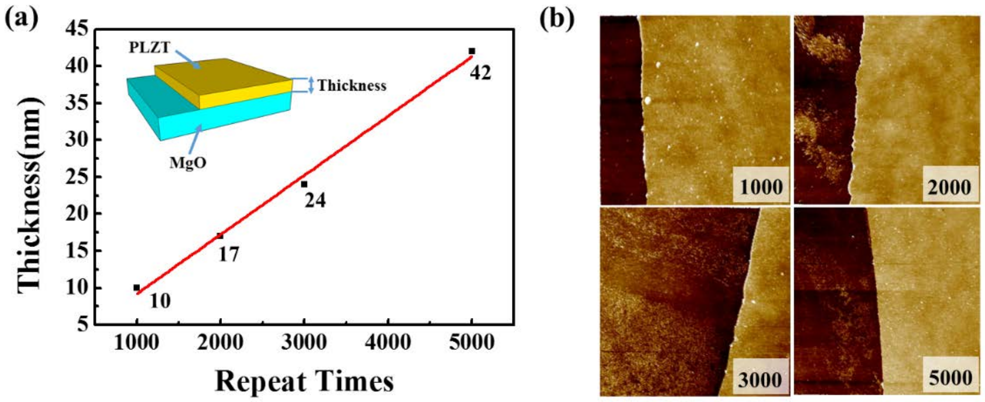


**Figure S3.** (a) Calibration curves for the growth rate of the PLZT thin films on MgO (200). (b) Atomic force microscopy (AFM) images of steps for samples deposited with different repetition times of the excitation laser: 1000, 2000, 3000 and 5000.

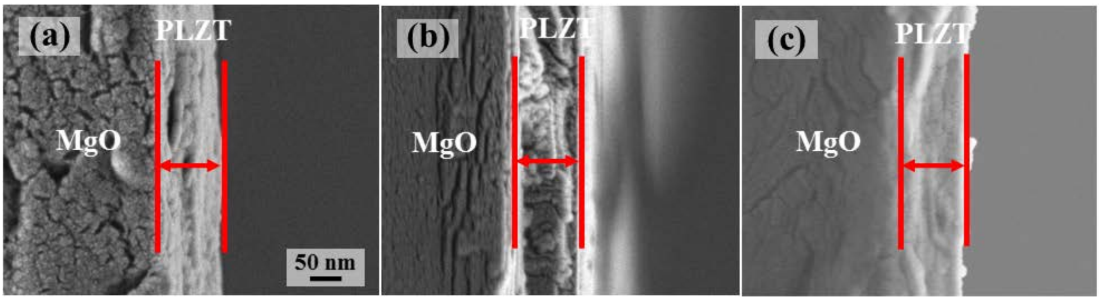


**Figure S4.** SEM cross-sectional views of the PLZT thin film fabricated at various temperatures: (a) 650 (b) 700 and (c) 750 °C.

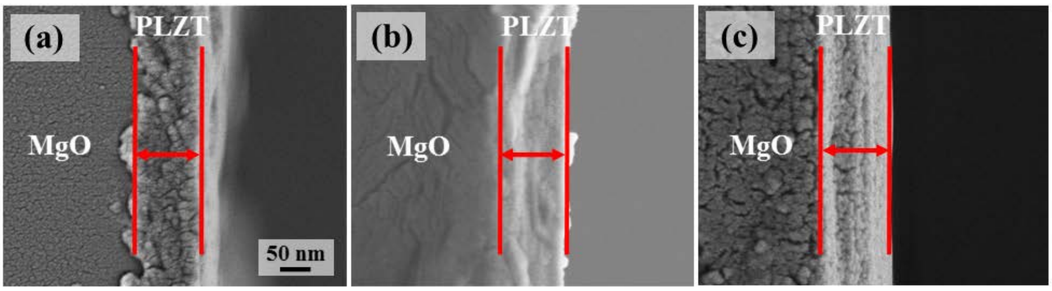


**Figure S5.** SEM cross-sectional views of the PLZT thin films annealed at various oxygen pressure: (a) 30 (b) 50 and (c) 100 Pa.

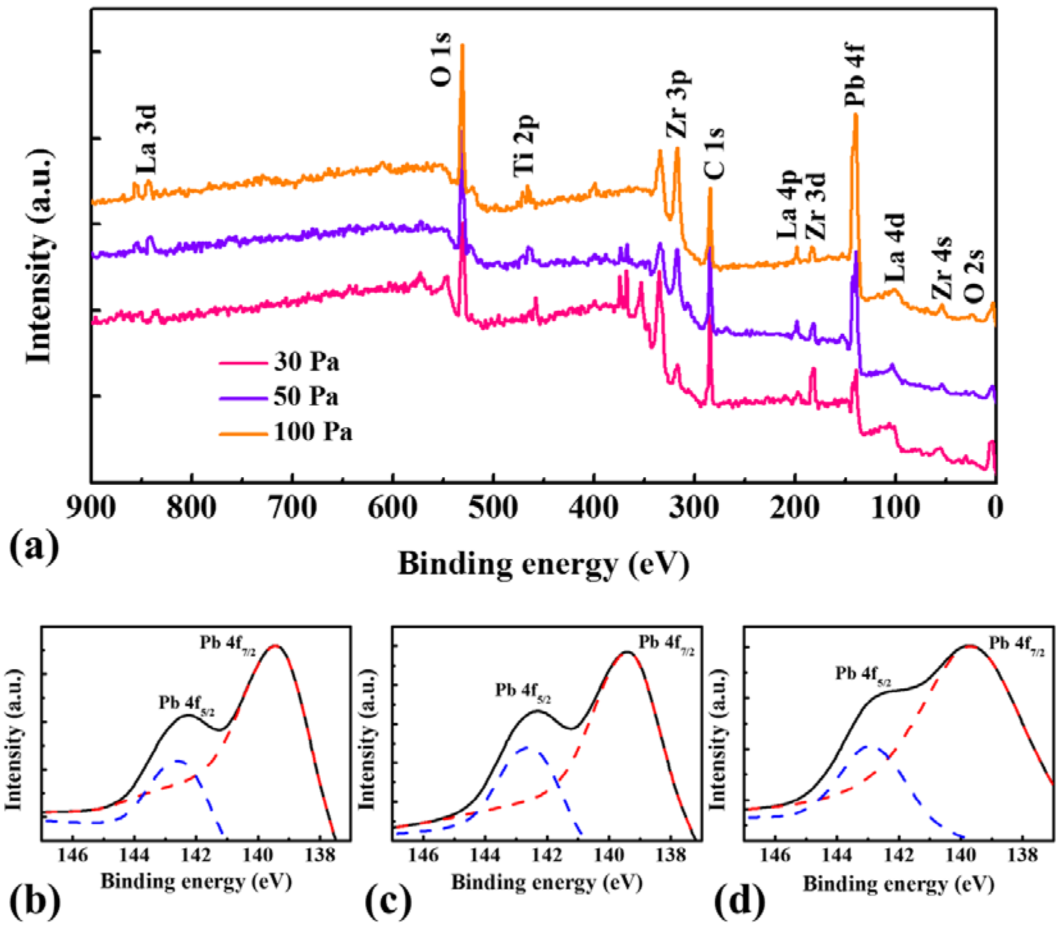


**Figure S6.** (a) X-Ray photoelectron spectroscopy (XPS) spectra of the PLZT thin films annealed at various oxygen pressure: 30, 50, 100 Pa. Core level spectra of Pb 4f for each sample: (b) 30 (c) 50 and (d) 100 Pa

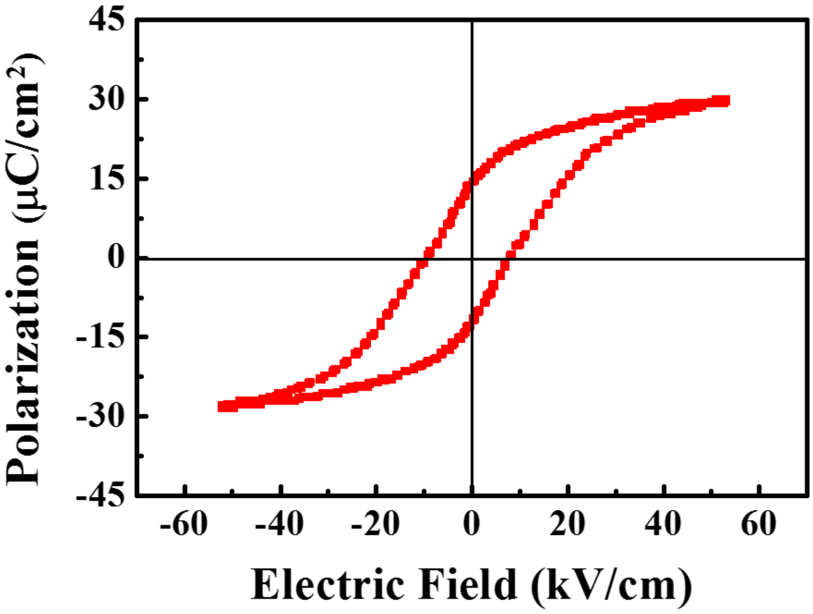


**Figure S7.** The P-E loop of the PLZT target.

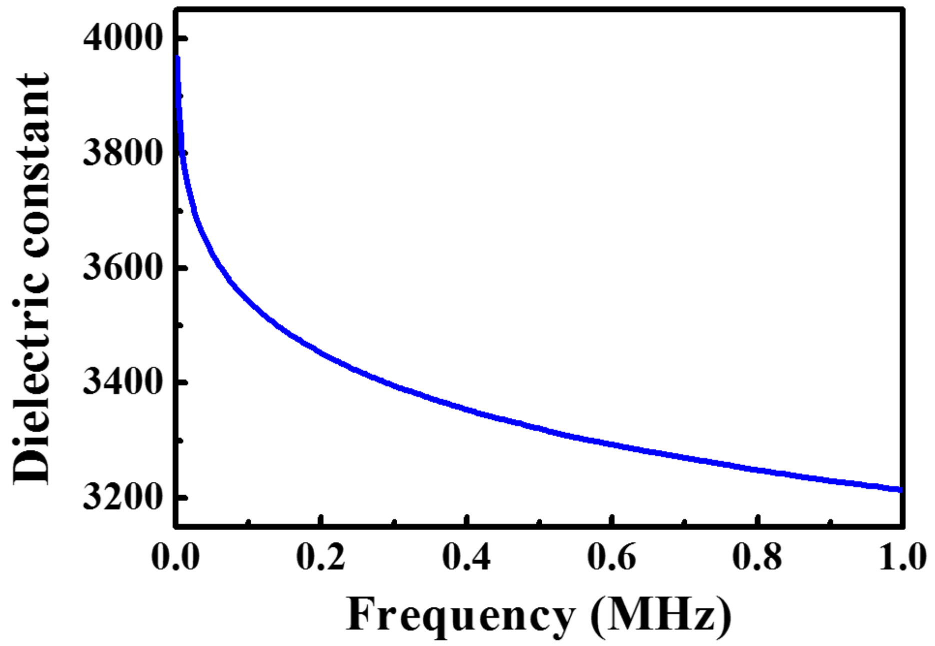


**Figure S8.** The room-temperature frequency dependent dielectric constant of the PLZT target.

**Table S1.** Binding energy peak positions of the core levels of Pb for the PLZT thin films annealed at various oxygen pressure:

| Annealing oxygen pressure | Pb $4f_{5/2}$ | Pb $4f_{7/2}$ |
|---|---|---|
| 30 Pa | 142.5 | 139.2 |
| 50 Pa | 142.6 | 139.4 |
| 100 Pa | 142.8 | 139.6 |

**Table S2.** The dielectric constant of the PLZT thin films at each bias voltages with the fixed frequency of 1kHz.

| **Bias (v)** | 0.2 | 0.4 | 0.6 | 0.8 | 1.0 |
|---|---|---|---|---|---|
| **Dielectric constant** | 1573 | 1523 | 1474 | 1438 | 1425 |
